# Supplementary material for: Unveiling the Bioactive Potential of the Invasive Jellyfish Phyllorhiza punctata Through Integrative Transcriptomic and Proteomic Analyses
Source: Biomolecules. 2025 Aug 4;15(8):1121. doi: 10.3390/biom15081121 (PMC12383608; doi:10.3390/biom15081121)
Supplement: Supplementary file 1 [file biomolecules-15-01121-s001.zip › Suplementary Material/FileS3/PEPFOLD4/XP_065067357_5146/clusters.html]

 PEP-FOLD clusters


Download archive of all models  here   

|  |  |  |
| --- | --- | --- |
| # | file | sOPEP |
| # cluster 1 - Size: 7 |  |  |
|  | XP\_065067357\_5146-model1 | -15.8487 |
|  | XP\_065067357\_5146-model1.1 | -15.7791 |
|  | XP\_065067357\_5146-model1.2 | -15.7469 |
|  | XP\_065067357\_5146-model1.3 | -15.7469 |
|  | XP\_065067357\_5146-model1.4 | -15.5881 |
|  | XP\_065067357\_5146-model1.5 | -15.5721 |
|  | XP\_065067357\_5146-model1.6 | -15.5599 |
| # cluster 2 - Size: 8 |  |  |
|  | XP\_065067357\_5146-model2 | -15.8275 |
|  | XP\_065067357\_5146-model2.1 | -15.8114 |
|  | XP\_065067357\_5146-model2.2 | -15.7947 |
|  | XP\_065067357\_5146-model2.3 | -15.6894 |
|  | XP\_065067357\_5146-model2.4 | -15.6283 |
|  | XP\_065067357\_5146-model2.5 | -15.5998 |
|  | XP\_065067357\_5146-model2.6 | -15.5601 |
|  | XP\_065067357\_5146-model2.7 | -15.5364 |
| # cluster 3 - Size: 31 |  |  |
|  | XP\_065067357\_5146-model3 | -15.735 |
|  | XP\_065067357\_5146-model3.1 | -15.735 |
|  | XP\_065067357\_5146-model3.2 | -15.6922 |
|  | XP\_065067357\_5146-model3.3 | -15.6922 |
|  | XP\_065067357\_5146-model3.4 | -15.6922 |
|  | XP\_065067357\_5146-model3.5 | -15.6549 |
|  | XP\_065067357\_5146-model3.6 | -15.6549 |
|  | XP\_065067357\_5146-model3.7 | -15.6549 |
|  | XP\_065067357\_5146-model3.8 | -15.6549 |
|  | XP\_065067357\_5146-model3.9 | -15.6548 |
|  | XP\_065067357\_5146-model3.10 | -15.6548 |
|  | XP\_065067357\_5146-model3.11 | -15.654 |
|  | XP\_065067357\_5146-model3.12 | -15.6512 |
|  | XP\_065067357\_5146-model3.13 | -15.6505 |
|  | XP\_065067357\_5146-model3.14 | -15.6505 |
|  | XP\_065067357\_5146-model3.15 | -15.6326 |
|  | XP\_065067357\_5146-model3.16 | -15.6326 |
|  | XP\_065067357\_5146-model3.17 | -15.6302 |
|  | XP\_065067357\_5146-model3.18 | -15.6082 |
|  | XP\_065067357\_5146-model3.19 | -15.6038 |
|  | XP\_065067357\_5146-model3.20 | -15.6038 |
|  | XP\_065067357\_5146-model3.21 | -15.6038 |
|  | XP\_065067357\_5146-model3.22 | -15.6038 |
|  | XP\_065067357\_5146-model3.23 | -15.6038 |
|  | XP\_065067357\_5146-model3.24 | -15.6038 |
|  | XP\_065067357\_5146-model3.25 | -15.6038 |
|  | XP\_065067357\_5146-model3.26 | -15.6038 |
|  | XP\_065067357\_5146-model3.27 | -15.6007 |
|  | XP\_065067357\_5146-model3.28 | -15.531 |
|  | XP\_065067357\_5146-model3.29 | -15.531 |
|  | XP\_065067357\_5146-model3.30 | -15.531 |
| # cluster 4 - Size: 7 |  |  |
|  | XP\_065067357\_5146-model4 | -15.7277 |
|  | XP\_065067357\_5146-model4.1 | -15.6768 |
|  | XP\_065067357\_5146-model4.2 | -15.6768 |
|  | XP\_065067357\_5146-model4.3 | -15.6672 |
|  | XP\_065067357\_5146-model4.4 | -15.6672 |
|  | XP\_065067357\_5146-model4.5 | -15.5557 |
|  | XP\_065067357\_5146-model4.6 | -15.5557 |
| # cluster 5 - Size: 13 |  |  |
|  | XP\_065067357\_5146-model5 | -15.7139 |
|  | XP\_065067357\_5146-model5.1 | -15.6883 |
|  | XP\_065067357\_5146-model5.2 | -15.6883 |
|  | XP\_065067357\_5146-model5.3 | -15.6883 |
|  | XP\_065067357\_5146-model5.4 | -15.6407 |
|  | XP\_065067357\_5146-model5.5 | -15.6063 |
|  | XP\_065067357\_5146-model5.6 | -15.6063 |
|  | XP\_065067357\_5146-model5.7 | -15.6063 |
|  | XP\_065067357\_5146-model5.8 | -15.6063 |
|  | XP\_065067357\_5146-model5.9 | -15.6063 |
|  | XP\_065067357\_5146-model5.10 | -15.5873 |
|  | XP\_065067357\_5146-model5.11 | -15.5873 |
|  | XP\_065067357\_5146-model5.12 | -15.5808 |
| # cluster 6 - Size: 6 |  |  |
|  | XP\_065067357\_5146-model6 | -15.7085 |
|  | XP\_065067357\_5146-model6.1 | -15.6946 |
|  | XP\_065067357\_5146-model6.2 | -15.6834 |
|  | XP\_065067357\_5146-model6.3 | -15.6788 |
|  | XP\_065067357\_5146-model6.4 | -15.6675 |
|  | XP\_065067357\_5146-model6.5 | -15.6398 |
| # cluster 7 - Size: 6 |  |  |
|  | XP\_065067357\_5146-model7 | -15.7049 |
|  | XP\_065067357\_5146-model7.1 | -15.7049 |
|  | XP\_065067357\_5146-model7.2 | -15.6945 |
|  | XP\_065067357\_5146-model7.3 | -15.6658 |
|  | XP\_065067357\_5146-model7.4 | -15.6637 |
|  | XP\_065067357\_5146-model7.5 | -15.644 |
| # cluster 8 - Size: 9 |  |  |
|  | XP\_065067357\_5146-model8 | -15.6947 |
|  | XP\_065067357\_5146-model8.1 | -15.6947 |
|  | XP\_065067357\_5146-model8.2 | -15.6947 |
|  | XP\_065067357\_5146-model8.3 | -15.6947 |
|  | XP\_065067357\_5146-model8.4 | -15.6349 |
|  | XP\_065067357\_5146-model8.5 | -15.6161 |
|  | XP\_065067357\_5146-model8.6 | -15.5752 |
|  | XP\_065067357\_5146-model8.7 | -15.5752 |
|  | XP\_065067357\_5146-model8.8 | -15.5452 |
| # cluster 9 - Size: 2 |  |  |
|  | XP\_065067357\_5146-model9 | -15.6894 |
|  | XP\_065067357\_5146-model9.1 | -15.6771 |
| # cluster 10 - Size: 6 |  |  |
|  | XP\_065067357\_5146-model10 | -15.6714 |
|  | XP\_065067357\_5146-model10.1 | -15.6391 |
|  | XP\_065067357\_5146-model10.2 | -15.6391 |
|  | XP\_065067357\_5146-model10.3 | -15.6282 |
|  | XP\_065067357\_5146-model10.4 | -15.6148 |
|  | XP\_065067357\_5146-model10.5 | -15.6148 |
| # cluster 11 - Size: 5 |  |  |
|  | XP\_065067357\_5146-model11 | -15.6359 |
|  | XP\_065067357\_5146-model11.1 | -15.6359 |
|  | XP\_065067357\_5146-model11.2 | -15.6359 |
|  | XP\_065067357\_5146-model11.3 | -15.6359 |
|  | XP\_065067357\_5146-model11.4 | -15.6105 |
|  |  |  |
